# Comprehensive GWAS and Transcriptome Analysis Discovered Candidate Gene Associated with Starch Pasting Properties of Temperate *japonica* rice (*Oryza sativa* L.)

**DOI:** 10.1186/s12284-025-00782-8

**Published:** 2025-04-02

**Authors:** Yoon Kyung Lee, Su Jang, Jihwan Im, Hee-Jong Koh

**Affiliations:** 1 Royal Botanic Gardens, Kew, Kew Green, Richmond, Surrey TW9 3AE UK; 2 Department of Agriculture, Forestry and Bioresources, Seoul National University, Seoul, 08826 South Korea; 3 Seedling LLC, Dangjin, Chungnam South Korea

**Keywords:** *Oryza sativa* L., *Japonica* rice, *OsGLUTN*, Eating quality, Storage protein, RVA, Starch pasting, GWAS, RNAseq, Transcriptome analysis

## Abstract

The growing market demand for high eating quality (EQ) rice, driven by improved living standards, highlights the need to better understand its complex genetic architecture. Starch pasting properties are critical determinants of rice EQ, yet their genetic basis remains incompletely understood. This study aimed to unravel the genetic factors underlying starch pasting properties in temperate *japonica* rice panel of 284 accessions comprising landraces and improved varieties. Genome-wide association studies conducted for two years identified 59 significant lead SNPs. Among them, consistent lead SNPs on chromosomes 2 and 8, in addition to the well-characterized *Wx* gene on chromosome 6 were detected from multiple traits and years. LD block analysis and transcriptome analysis of accessions with extreme phenotypes identified *OsGLUTN* (Os02g0224300/LOC_Os02g13130), a gene encoding a high molecular weight glutenin subunit-like protein, as a strong candidate. Clustering patterns of differential gene expressions showed higher expression of *OsGLUTN* in low-viscosity cultivars and lower expression in high-viscosity cultivars. Haplotype analysis revealed significant variations in viscosity traits associated to *OsGLUTN* alleles, and functional validation using enhancer active tagging line showed significantly reduced starch viscosity, confirming its role in EQ regulation. While the *Wx* accounted for most viscosity traits, the identification of novel loci on chromosomes 2 and 8 highlights additional genetic factors to EQ variation. These findings deepen our understanding of how storage protein metabolism impacts rice grain quality. The identification of *OsGLUTN* provides a foundation for breeding programs focused on developing rice varieties with improved cooking and eating qualities, addressing growing consumer demand for premium rice.

**Supplementary Information:**

The online version contains supplementary material available at 10.1186/s12284-025-00782-8.

## Background

Rice (*Oryza sativa* L.) is a staple food, providing a major source of calories (Qian et al. 2016). Rice grain is mainly composed of starch, approximately 80–85% in endosperm (Champagne et al. 2004). It is not surprising that eating quality (EQ) of rice is affected by various starch related physico-chemical properties. On the other hand, EQ is a complicated trait which encompass all sensory attributes. Hence, all the major and minor contributing factors related to the EQ should be inclusively considered to understand the trait.

Starch metabolism has been extensively studied; ADP-glucose is transferred to amylopectin and amylose (Smith et al. 1997; Tetlow et al. 2004) by starch synthases like soluble starch synthases (SSI, SSII, SSIII) and granule-bound starch synthases (GBSSI, GBSSII). Starch-branching enzymes (SBEI, SBEII) catalyze the hydrolysis of α-1,4 glucosidic linkages and form α-1,6 glucosidic linkages and starch-debranching enzymes (ISA1, ISA2, PUL) determine the structure of amylopectin (Morell et al. 2003; Li et al. 2003; Slattery et al. 2000; Gao et al. 2011; Tetlow and Emes 2017). It is known that amylose content is directly related to EQ, and other starch properties like gel consistency, viscosity, and gelatinization temperature are also considered in determining EQ.

Starch pasting properties are one of the physical traits of starch granules which are quantitatively measured by rapid viscosity analysis (RVA). Pasting temperature measures the temperature which pasting is initiated, thus it can evaluate the cooking quality. When starch granules absorb moistures with heating, the viscosity is increased by swelling and reach its maximum viscosity. With the continuous rotation of spindle, the starch granules are broken into smaller particles and soluble components are redistributed, leading to decreased viscosity and reach the minimum viscosity. The soluble amylose and amylopectin get mixed upon cooling, and the viscosity gets slightly increased then reach its final viscosity when cooling at 50 °C is over. The breakdown viscosity is difference of maximum viscosity value and minimum viscosity value, implying the level of starch granule destruction. The setback viscosity is a difference of final viscosity value and maximum viscosity value, measuring the level of starch aging. It is generally known that high maximum and breakdown viscosity, and low setback viscosity contributes to good EQ. Recent QTLs studies identified genetic regions contributing the starch pasting properties using mapping populations. Major QTLs were identified from RVA (Ponce et al. 2018) including *qCPV6*, *qPKV6*, and *qBDV6.1* nearby *Wx* gene. Also, natural alleles of major gene *ALK/SSIIa* lowering gelatinization temperature were identified (Chen et al. 2020). Most recently, a novel gene, *OsCRLK2*, located in *qGT6.4* was identified and verified its functions in rice quality (Chen et al. 2024).

To rapidly unravel quantitative traits like EQ, genome-wide association study has been widely adopted. GWAS of amylose and protein content using various sources of rice accessions have been conducted yet specifying and cloning other related genes was not successful than detecting *GBSSI* region as one of lead SNPs (Xu et al. 2016; Wang et al. 2017a; Bao et al. 2017). Many studies challenged to dissect the genetic bases of other EQ traits like grain appearance and milling quality (Wang et al. 2017b), cooked rice texture (Misra et al. 2018), starch viscosity (Buenafe et al. 2021), then complemented the candidate genes from GWAS with haplotype analyses, transcriptome analyses, and functional studies for verification.

## Materials and Methods

### Plant Materials

*Japonica* rice including improved cultivars and landraces which its heading date is within August was selected for comprising the panels. The panels composed of 196 and 281 accessions (Additional file 1: Table S1) were cultivated in an experimental field at Seoul National University, Suwon, South Korea in 2019 and 2020, respectively. The general lowland cultivation method was applied.

Rice seeds with 35 S activation-tagged T-DNA insertion in candidate gene (*OsGLUTN*-D; PFG_3A-02504.L) and the parental cultivar “Dongjin” were acquired from Crop Biotech Institute at Kyung Hee University, South Korea (Jeong et al. 2002; Ryu et al. 2004; An et al. 2005). All plants were grown in the field with general lowland cultivation method in 2021.

### Phenotypic Analyses

Starch pasting properties were measured using Rapid Visco Analyzer (Newport Scientific, Warriewood, Australia) in 2019 and 2020 for year replicates of GWAS and in 2021 using T-DNA lines. In brief, the heading date of each accession was recorded, and plants were harvested when reaching 45–50 days after heading. The harvested plants were air dried until reaching the grain moisture content of 13–14%, and subsequently threshed using a thresher. The grains were dehulled (using a dehull machine), milled to 92.2% (using a milling machine), and stored at 12 °C storage-room. Finely ground polished rice flour (4 g) was added to 15 ml of distilled water, spindle speed and temperature setting followed the method described in the AACC Method 61 − 02 (American Association of Cereal Chemists, 2000); heating cycle (50–95 °C) - hold (95 °C) - cooling cycle (95–50 °C). Maximum viscosity, minimum viscosity, final viscosity, setback viscosity, and breakdown viscosity were measured in Rapid Viscosity Unit (RVU). Moreover, peak time (in mins) and pasting temperature (in °C) were measured as well.

### Genotypic Data

Total DNA was extracted from young leaves of 6-week-old plants of each accession according to the CTAB method (Murray and Thompson 1980). Sheared DNA into 450–500 bp fragments were used for preparing DNA library using TruSeq Nano DNA Library Prep kits (Illumina, San Diego, CA, USA) following the instructions of the manufacturer’s protocol. The constructed DNA library was sequenced using an Illumina HiSeq X system and obtained 2 × 150 bp paired end reads with sequencing depth of > 10× for each sample. The adaptors were removed from the raw reads, and low-quality bases were eliminated using Trimmomatic v0.38 (Bolger et al. 2014). Quality-trimmed reads were mapped against rice reference genome, Nipponbare IRGSP v1.0, using bwa-mem with default parameters of BWA software v0.7.17 (Li and Durbin 2009). The mapped reads were sorted using Samtools v1.9 (Li et al. 2009) and removed the duplicates using Picard v2.20.2 (http://broadinstitute.github.io/picard/). Nucleotide variants were called using HaplotypeCaller function of GATK v4.1.2 (McKenna et al. 2010). The heterozygous genotypes were filtered out. After removing the variants with missing rates > 0.20 and minor allele frequency < 0.05, 1,254,682 SNPs were identified.

### Association Analysis and Candidate Gene Identification

GWAS was performed using Factored Spectrally Transformed Linear Mixed Model using FastLmm v2.07 (Lippert et al. 2011). The outputs of association analysis subsequently identified lead SNP loci in the regions that exhibit significant association with trait variation at highest R^2^ and lowest false discovery rate adjusted P-values (threshold 0.01 and 0.05). Manhattan plots and Q-Q plots for each trait were generated using “rMVP” in the R package (Yin et al. 2021). Single-SNP associations were considered true positive when a peak of multiple SNPs was detected at lower -log10 (P-values) in the Manhattan plot. For identification of the candidate genes for each starch pasting property, the LD heatmaps in the vicinity of the peaks in Manhattan plots were constructed using “LDBlockShow” (Dong et al. 2021), the LD was measured in R^2^, and the blocks were detected using PLINK method.

### RNAseq and Transcriptome Analysis

Accessions that showed extreme values in RVA were selected; J10-“Jaeraeryukdo”, J301-“Cheonggunbyeo”, J230-“Mojo”, J336-“Gopum”, J214-“Younghojinmi”, J361-“Chiyominori”. Total RNA was extracted from developing endosperms at 7–9 days after heading while rice hulls and embryos were removed. Each of two samples per accession was composed of about 6–8 rice endosperms. In total, 12–15 endosperms were used in RNA extraction of the accession. TaKaRa MiniBEST Plant RNA Extraction Kit (Takara, Japan) was used following the protocol of manufacturer. RNA sequencing was performed using size-selected and quality-checked samples generated from TruSeq Stranded mRNA Sample Prep Kit by Illumina NovaSeq 6000 platform for 150 bp paired-end reads. About 6 Gb of outputs were generated for each sample. Generated raw sequences of samples were processed for adaptor trimming and quality trimming using Trimmomatic v0.38. The trimmed fastq files were subjected for quality check using FastQC v0.11.9 (Andrews 2010). The sequences were aligned to Nipponbare reference sequence (IRGSP-1.0) using HISAT2 v2.2.1 (Kim et al. 2019) and generated the output into SAM files. Using Samtools v1.9, the SAM files were converted into BAM files and indexed for further analyses. The mapped sequencing reads were aligned to IRGSP-1.0 transcripts file using featureCounts (Liao et al. 2014) function of R, and the genomic features were counted. The read counts were generated, and differential gene expressions were normalized using edgeR (Robinson et al. 2010) function with CPM method. The TMM normalized log CPM values of genes for the samples were subjected for clustering analysis using Clust (Abu-Jamous and Kelly 2018), and clusters of genes that are consistently co-expressed were identified. The gene lists of identified clusters were used in gene ontology enrichment analysis using PANTHER database v17.0 (Thomas et al. 2003, 2006) for biological process and molecular function. The ontology was visualized using REVIGO (Supek et al. 2011) with removing the redundant GO terms by clustering and reduction algorithms.

### PCR Analysis

Genomic PCR was performed with the combinations of left and right gene specific primers, and T-DNA plasmid pGA2715 left border primer (Additional file 1: Table S2). PCR amplification was performed in 20 µL reaction mixtures containing 100 ng template DNA, 0.5 U Prime Taq polymerase (GeNet Bio, South Korea), 1 × PCR buffer, 0.5 µM of each primer. The PCR thermal cycle was 95 °C for 5 min; 35 cycles of 95 °C for 30 s, optimum primer annealing temperature for 30 s, and 72 °C for 40 s; and final extension at 72 °C for 5 min.

Total RNA of *OsGLUTN*-D and it’s wild type, “Dongjin”, was extracted from 7 to 9 days old developing endosperms as the same method used for RNA-seq using TaKaRa MiniBEST Plant RNA Extraction Kit (Takara, Japan) following the manufacturer’s protocol. First-strand cDNA of the samples were synthesized using AccuPower ^®^ Rocketscript Cycle RT Premix (Bioneer, South Korea), and qPCR was performed using AccuPower^®^ 2X GreenStar Master Mix (Bioneer, South Korea) on a Exicycler™ 384 Real-Time Quantitative Thermal Block (Bioneer, South Korea) according to the manufacturer’s instructions using the designed primers (Additional file 1: Table S2). The expression levels of T-DNA insertion line and the wild type for *OsGLUTN* were relatively compared by normalizing each with *Ubiquitin-5*, a housekeeping gene, and applying delta-delta CT method. Fold change for expression level were compared using two-tailed Student’s t-test.

### Statistical Analyses

PCA was performed using pca function of PLINK v1.9 (Chang et al. 2015; www.cog-genomics.org/plink/1.9/), and correlation matrix was computed using cor function of R package. All the figures were generated using ggplot2 R package. All statistical analysis was performed using R v3.5.3.

## Results

### Panel Composition and Phenotypic Variations

**Fig. 1:**
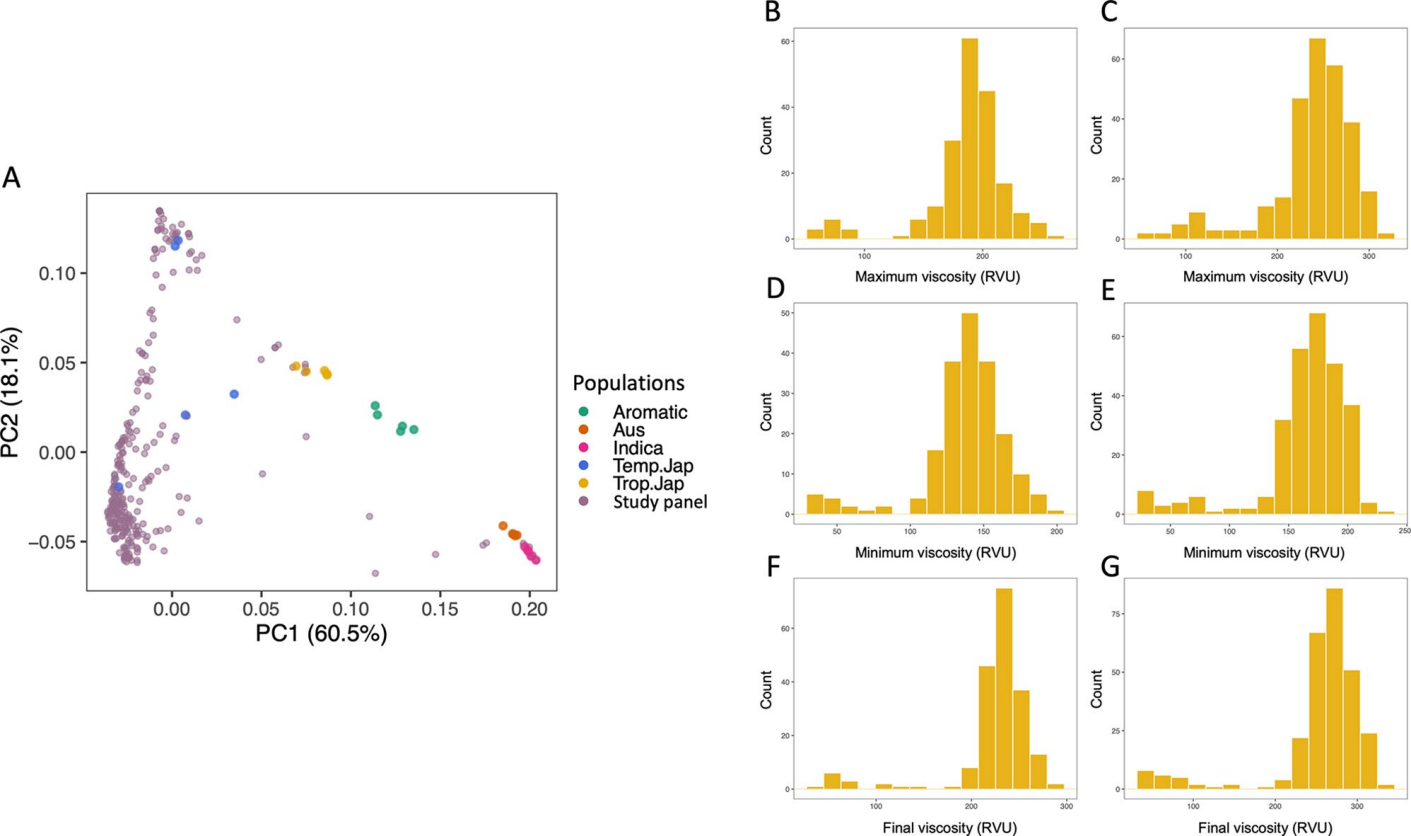
PCA and histogram of starch pasting properties of the accessions. (**A**) PCA of the panel compared with 5 subpopulations as check variety, color-coded dots indicate each of accession. Histogram of observed phenotypes (**B**) maximum viscosity in 2019, (**C**) maximum viscosity in 2020, (**D**) minimum viscosity in 2019, (**E**) minimum viscosity in 2020, (**F**) final viscosity in 2019, and (**G**) final viscosity in 2020

In the current study, total of 284 rice accessions were used. The rice panel was composed of 171 improved varieties and 113 landraces. Before conducting genetic analyses, the accessions were studied for its population analysis. Principal component analysis (PCA) of the accessions with 25 check varieties (Additional file 1: Table S3, 5 accessions each of aromatic, *aus*, *indica*, temperate *japonica*, and tropical *japonica* rice) identified most of the accessions as temperate *japonica* (Fig. 1A) while PC1 explained 60.5% of the variations, and PC2 explained 18.1% of the variations (Additional file 6: Fig. S5). The subpopulation of 284 accessions classified as temperate *japonica* rice was used for further analysis.

To assess the starch pasting properties, RVA was conducted for 2 years, 2019 and 2020. The histograms of phenotypic traits of each year (Fig. 1B-G and Additional file 2: Fig. S1) mostly showed normal distributions, suggesting that the traits are related to and appropriate for the qualitative trait analyses. Among the pasting properties, maximum viscosity showed strong positive correlations to minimum viscosity, final viscosity, and breakdown viscosity, while negative correlations to setback viscosity and pasting temperature (Additional file 3: Fig. S2).

### Genome-wide Association Analysis

**Fig. 2:**
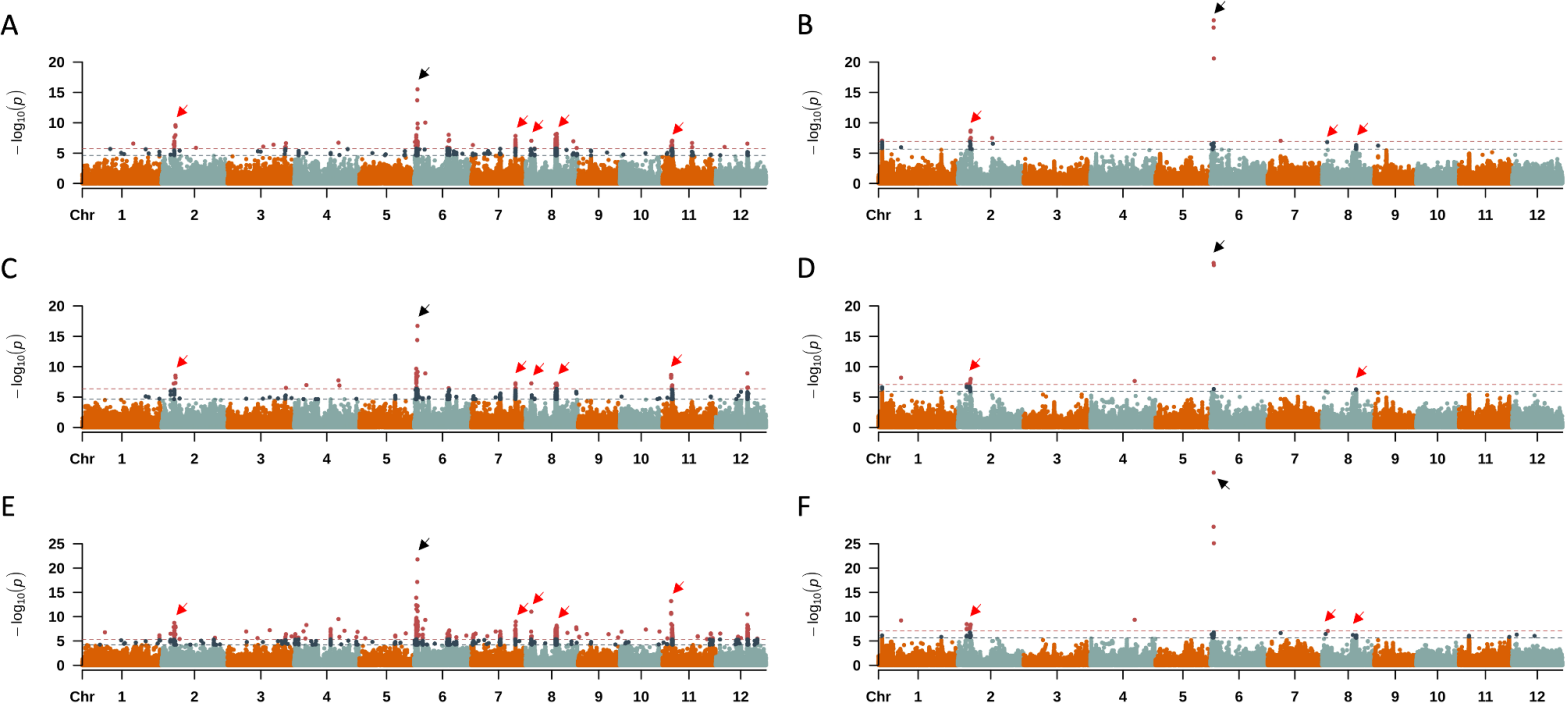
Manhattan plots of starch pasting properties for 2-year replicates. (**A**) maximum viscosity in 2019, (**B**) maximum viscosity in 2020, (**C**) minimum viscosity in 2019, (**D**) minimum viscosity in 2020, (**E**) final viscosity in 2019, and (**F**) final viscosity in 2020. The blue dots above blue dashed lines indicate the SNPs above the lowest false discovery rate adjusted P-values were 0.05. The red dots above red dashed lines indicate < the threshold of 0.01. Black arrows indicate the position of *Wx* region, while red arrows indicate commonly detected genetic regions

A total of 7 traits related to rice starch pasting properties were measured in 2 year-replicate. From a panel composed of 196 accessions in 2019, *Wx* was commonly detected from the Manhattan plots of all traits except for breakdown viscosity (Fig. 2A, C,E and Additional file 4: Fig. S3). Moreover, lead SNPs on chromosomes 2, 7, 8, and 11 were commonly detected from maximum viscosity, minimum viscosity, and final viscosity. In 2020 panel composed of 281 accessions, *Wx* was similarly detected from all the 7 starch pasting properties (Fig. 2B, D,F and Additional file 4: Fig. S3). Common lead SNPs were also detected from chromosomes 2 and 8 for maximum viscosity, minimum viscosity, and final viscosity (Additional file 1: Table S4). In conclusion, the novel lead SNPs in chromosomes 2 and 8 were commonly detected from 2-year replicates of maximum viscosity, minimum viscosity, and final viscosity. The lead SNPs that were significantly associated with starch pasting properties contained 162 genes which molecular functions are highly related to grain compositions, starch and protein metabolism related, and carbohydrate and lipid metabolic processes (Additional file 1: Table S5).

### Transcriptome Analysis

To further narrow down the associated genes for starch pasting properties, RNA sequencing and transcriptome analysis of accessions with extreme phenotypes were conducted. J10, J301, and J230 were selected for all having lowest maximum, minimum, and final viscosities, while J336, J214, and J361 were selected for having highest values in maximum, minimum, and final viscosities. The average maximum, minimum, and final RVU of the low group measured in 2020 were 200.06, 145.41, and 240.93, respectively. Those of the high group were 282.08, 182, and 281.47, respectively.

**Fig. 3:**
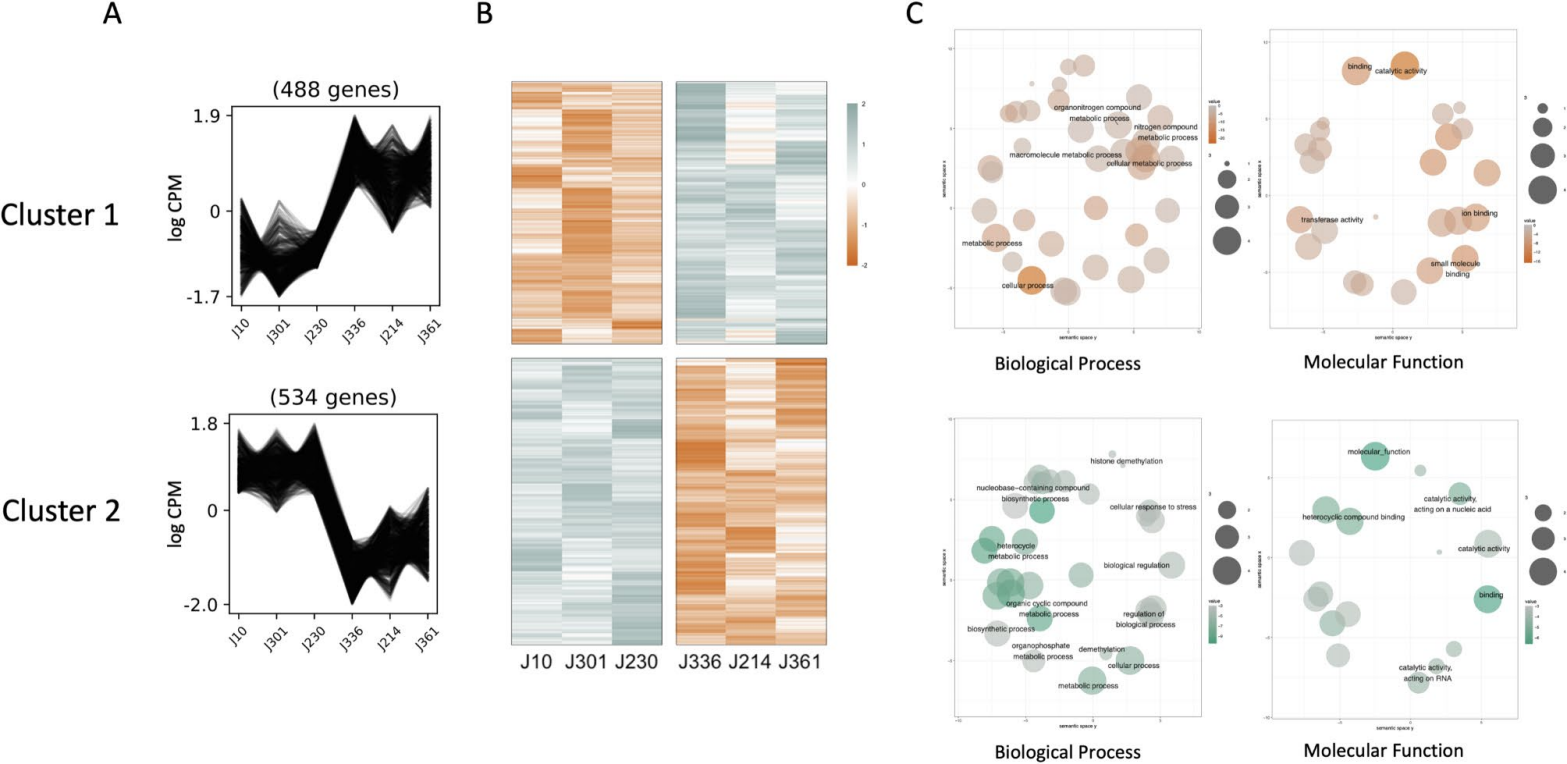
Transcriptome analysis of cultivars showing extreme RVA traits. (**A**) Clustering analysis of differentially expressed genes, (**B**) heatmap analysis of the gene expression levels of the cultivars, which orange color to green color indicate from − 2 fold change to + 2, and (**C**) GO enrichment analysis, panels of gene sets with orange to grey shades are for cluster 1 and those of green to grey shades are for cluster 2. The circle size indicates number of genes composing gene sets, while the color intensity implies the intensity

The log values of normalized read counts were obtained, and clustering analysis identified groups of genes that co-segregate with the phenotypes. Cluster 1 was composed of 488 genes which showed low logCPM values from low RVA cultivars and high logCPM values from high RVA cultivars (Fig. 3A). Cluster 2 was composed of 534 genes with high logCPM values from low RVA cultivars and low logCPM values from high RVA cultivars. The genes constitute these clusters showed clearly different expression patterns in the heatmap of the cultivars (Fig. 3B). The gene ontology enrichment analysis was performed for each cluster (Fig. 3C). The major biological process of genes from cluster 1 were cellular process and metabolic process like macromolecule metabolic process, cellular metabolic process, nitrogen compound metabolic process, and organonitrogen compound metabolic process. The identified molecular functions of the genes were catalytic activity, transferase activity, ion binding, and small molecule binding. In case of cluster 2, the biological processes of genes in the cluster were mainly metabolic process and cellular process, like heterocycle metabolic process, organic cyclic compound metabolic process, and nucleobase-containing compound biosynthetic process. The molecular functions of the genes were identified as heterocyclic compound binding, catalytic activity acting on nucleic acid etc.

### Candidate Gene Analysis

**Fig. 4:**
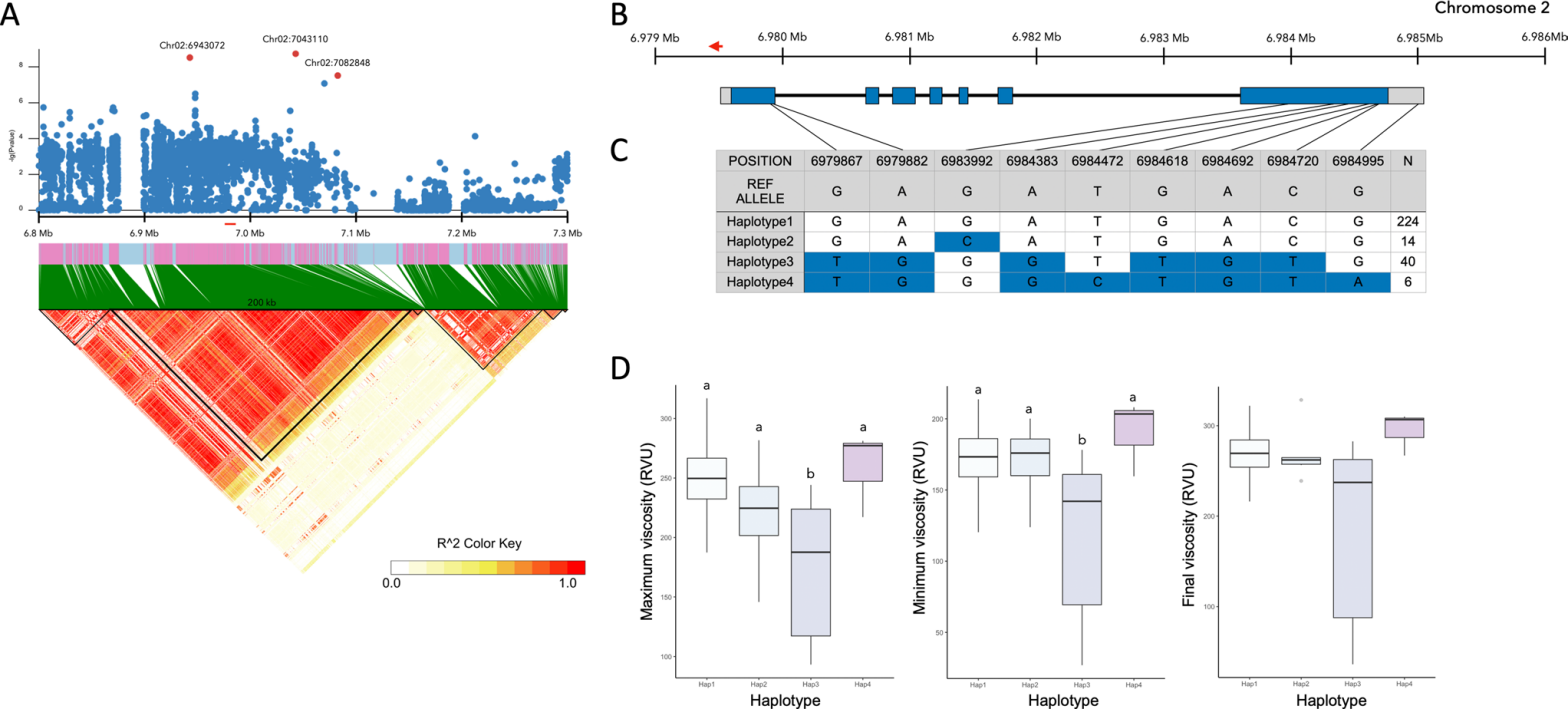
The candidate gene analysis associated with starch pasting properties. (**A**) Regional association and LD heat map of the candidate region, red dots indicate the significant SNPs and short red line on genome indicate the position of *OsGLUTN* in chromosome 2, (**B**) gene structure of *OsGLUTN* (LOC_Os02g13130.1), red arrow on genome indicates the position of T-DNA insertion, (**C**-**D**) haplotype analysis with the allelic variations in the gene that cause missense mutation. Starch pasting properties of each haplotype was compared using RVA. Significance difference between groups indicate Tukey’s post-hoc test

The list of genes from cluster analysis were compared with the genes within LD blocks (Fig. 4A) of commonly detected lead SNPs. The candidate gene for rice starch pasting properties was narrowed down to *OsGLUTN* (Os02g0224300/LOC_Os02g13130), which belonged to cluster 2. The nucleotide of *OsGLUTN* (LOC_Os02g13130.1) is 2,100 bp in length composed of 7 coding exons, located in short arm of chromosome 2 from 6,979,511 bp to 6,985,051 bp in positive strand, with predicted protein length of 700 amino acids (Fig. 4B). The annotated function of the gene is high molecular weight glutenin family in RAP-DB. Using the SNP variants of the study panel, functional and non-functional alleles in gene region were identified (Fig. 4C). The haplotypes of 6 accessions used for gene clustering analysis were all Haplotype1 except for J230 which was Haplotype3. Among the 4 haplotypes, haplotype 3 showed significantly lower maximum and minimum viscosity (Fig. 4D).

The mutant (*OsGLUTN*-D) with 35 S activation-tagged T-DNA insertion in the promoter region of candidate gene was obtained, and the insertion was confirmed using PCR analysis of HPT marker and combinations of gene specific markers and border marker of vector sequence (Additional file 5: Fig. S4A). The relative gene expression of *OsGLUTN*-D showed 2.34 times higher fold change compared to its wild type (Additional file 5: Fig. S4B). To demonstrate gene function, the RVA of Dongjin and *OsGLUTN*-D were measured and compared for starch pasting properties (Table 1). As a result, the maximum viscosity, minimum viscosity, final viscosity, and setback viscosity were significantly lower in *OsGLUTN*-D. While the breakdown viscosity was slightly increased, the T-DNA insertion affected the overall pasting properties.

**Table 1:** The comparison of pasting properties of T-DNA line and wild-type using RVA

	Maximum viscosity (RVU)	Minimum viscosity (RVU)	Final viscosity (RVU)	Breakdown viscosity (RVU)	Setback viscosity (RVU)	Peak time (min)	Pasting temp. (°C)
Dongjin	189.50	129.10	218.56	60.40	29.06	6.32	86.78
*OsGLUTN*-D	174.67^**^	108.02^**^	193.0^**^	66.65	18.33^**^	6.22	86.39

^**^Significance level at 0.01 of ANOVA test

## Discussion

Several major genes and QTLs are known to be related to the eating quality of rice. Most prominently, genes for starch biosynthesis such as *GBSS I*, branching enzymes, debranching enzyme, and starch synthases (Morell et al. 2003; Li et al. 2003; Slattery et al. 2000; Tetlow and Emes 2017) play pivotal role by controlling amylose content. On the other hand, protein content is related to the hardness or toughness of cooked rice. However, considering that major genes related to the amylose and protein content cannot explain all the EQ variations, seeking other major or minor genetic factors related to the EQ properties are essential in unraveling the architecture of EQ and controlling the trait.

Starch pasting property is closely related to the cooking properties and affect eating quality of rice. Previous studies on GWAS of starch paste viscosity properties for non-glutinous rice revealed QTLs nearby *isoamylase 3* gene in chromosome 9 and *starch synthase IV-1* gene in chromosome 1 (Xu et al. 2016). Although several studies revealed how the starch pasting and viscosity properties affect the textures, grain rigidity, and cooking quality (Balet et al. 2019; Pang et al. 2016; Yan et al. 2005; Bao et al. 2000), causal genes for variations of the property are nearly identified except for *Wx* in chromosome 6. *Wx* region was detected in most of the viscosity properties in this study as well, there were also consistently detected QTLs in other regions like chromosome 2 and 8.

Hundreds of genes were narrowed down by comprehensively comparing GWAS, LD block analysis, and differential expression clustering analysis. The identified candidate gene, *OsGLUTN*, exhibited high gene expression levels in the cultivars with low maximum, minimum, and final viscosity units, and low gene expression levels in the cultivars with high maximum, minimum, and final viscosity units. This pattern was also exhibited from the activation tagging lines that gain-of-function mutation showed significantly lower RVA values than the wild type. Moreover, haplotype analysis revealed different allele type led to significant differences in maximum and minimum viscosity. Based on the study of Likitwattanasade and Hongsprabhas (2010), a sample with protein reduction treatment showed high starch pasting properties, while it was decreased when protein content increased. In the confocal images of cooked rice, there was difference in the honeycomb structure, which is a complex of protein and starch, depending on the level of protein content.

The candidate gene, *OsGLUTN*, is annotated to encode high molecular weight glutenin subunit x-like protein, one of the storage proteins. A recent study by Li et al. (2022) revealed *OsPHYB* mutation led to down-regulation of storage protein genes including *OsGLUTN*, *Os02g0456100*, *OsEnS-51*, *Os06g0507150*, and explained it for the increase in the chalkiness of the mutant grains. Multiple genes are reported to be involved in protein metabolism up to date, and it is closely related to the chalkiness of grains (Xie et al. 2021). Also, regulation of the contents of amino acids and storage proteins could affect the quality of rice (Guo et al. 2020; Lin et al. 2017; Lu et al. 2018).

High molecular weight glutenin subunits are storage gluten proteins present in the starchy endosperm cells of wheat grain (Li et al. 2021). In hexaploidy wheat, *Glu-1* loci are known to encode high molecular weight glutenin subunits (Payne et al. 1984; Lawrence and Shepherd 1981) and highly related to end use quality. Moreover, the mechanism regulating the expression of this high molecular weight glutenin subunit is unclear up to date. In rice, an attempt had been made to exert wheat glutenin subunits accumulate in the endosperm of transgenic rice seeds (Jo et al. 2017). Thus, finding of this study on the association of viscosity and *OsGLUTN* could disclose more about how seed storage protein content affect EQ.

## Conclusions

With the significance of identifying genetic architecture of eating quality and minor effect genetic factors, this study identified *OsGLUTN*, a gene encoding a high molecular weight glutenin subunit-like protein on chromosome 2, as a key determinant of starch pasting properties and EQ in temperate *japonica* rice. Using GWAS, LD block analysis, transcriptome analysis, and functional validation, the gene was shown to significantly influence maximum, minimum, and final viscosity traits, with haplotype analysis and T-DNA mutant experiments confirming its role. The gene’s expression correlated with differential viscosity levels, likely through its impact on the protein-starch matrix. While the *Wx* on chromosome 6 remains a major player, novel loci on chromosomes 2 and 8 were consistently associated with viscosity traits, expanding our understanding of rice EQ traits. These findings provide valuable insights into storage protein metabolism’s role in rice grain quality and present opportunities for breeding rice varieties with improved cooking and eating properties. Furthermore, the study present opportunities for targeted breeding strategies aimed at enhancing starch pasting properties and optimizing cooking and eating qualities. Future research may explore the regulatory mechanisms of *OsGLUTN* and its interactions with other starch and protein metabolism pathways to further unravel the complex genetic architecture of rice quality.

## Electronic Supplementary Material

Below is the link to the electronic supplementary material.

Supplementary Material 1

Supplementary Material 2: Figure S1 Histogram of other starch pasting properties. Including breakdown viscosity, setback viscosity, pasting temperature and peak time in 2019 and 2020 each.

Supplementary Material 3: Figure S2 Correlation analysis of the starch pasting properties measured from RVA in 2020. Circle size imply the strength of correlation, while color indicate blue as positive correlation and red as negative correlation between traits. Correlation coefficients with a significant difference of at most 0.05 are marked with the values. V stands for viscosity.

Supplementary Material 4: Figure S3 Manhattan plots of other starch pasting properties. Including breakdown viscosity, setback viscosity, pasting temperature and peak time in 2019 and 2020 each. The blue dots above blue dashed lines indicate the SNPs above the lowest false discovery rate adjusted P-values were 0.05. The red dots above red dashed lines indicate < the threshold of 0.01.

Supplementary Material 5: Figure S4 PCR and qRT-PCR of T-DNA line. (A) PCR confirming the T-DNA insertion, LP, RP, and LB stands for left gene specific primer, right gene specific primer, and pGA2715 left border primer, respectively. The 35S enhancer active tagging line, *OsGLUTN*-D, was compared with its wild type parental cultivar, Dongjin. (B) Relative gene expression level of wild type, Dongjin, and *OsGLUTN*-D. Significance level indicates **, *P* < 0.01.

Supplementary Material 6: Figure S5 Explained variances of the principal components. Contributing PCs on the x axis, and explained variances (%) on the y axis of the bar graph.

Supplementary Material 7

## Data Availability

All the sequencing data generated in this study were deposited in the Sequence Read Archive database (www.ncbi.nlm.nih.gov/sra) at NCBI (National Center for Biotechnology Information) under BioProject number PRJNA1003283.

## Abbreviations

ANOVA: Analysis of variance
EQ: Eating quality
GO: Gene ontology
GWAS: Genome-wide association study
HPT: Hygromycin phosphotransferase gene
LD: Linkage disequilibrium
LogCPM: Log counts per million
PCA: Principal component analysis
PCR: Polymerase chain reaction
QTLs: Quantitative trait loci
RNAseq: RNA sequencing
RVA: Rapid viscosity analysis
RVU: Rapid viscosity unit
SNP: Single nucleotide polymorphism
