# Supplementary figures and images for: Comprehensive GWAS and Transcriptome Analysis Discovered Candidate Gene Associated with Starch Pasting Properties of Temperate japonica rice (Oryza sativa L.)
Source: Rice (N Y). 2025 Apr 2;18:23. doi: 10.1186/s12284-025-00782-8 (PMC11961803; doi:10.1186/s12284-025-00782-8)

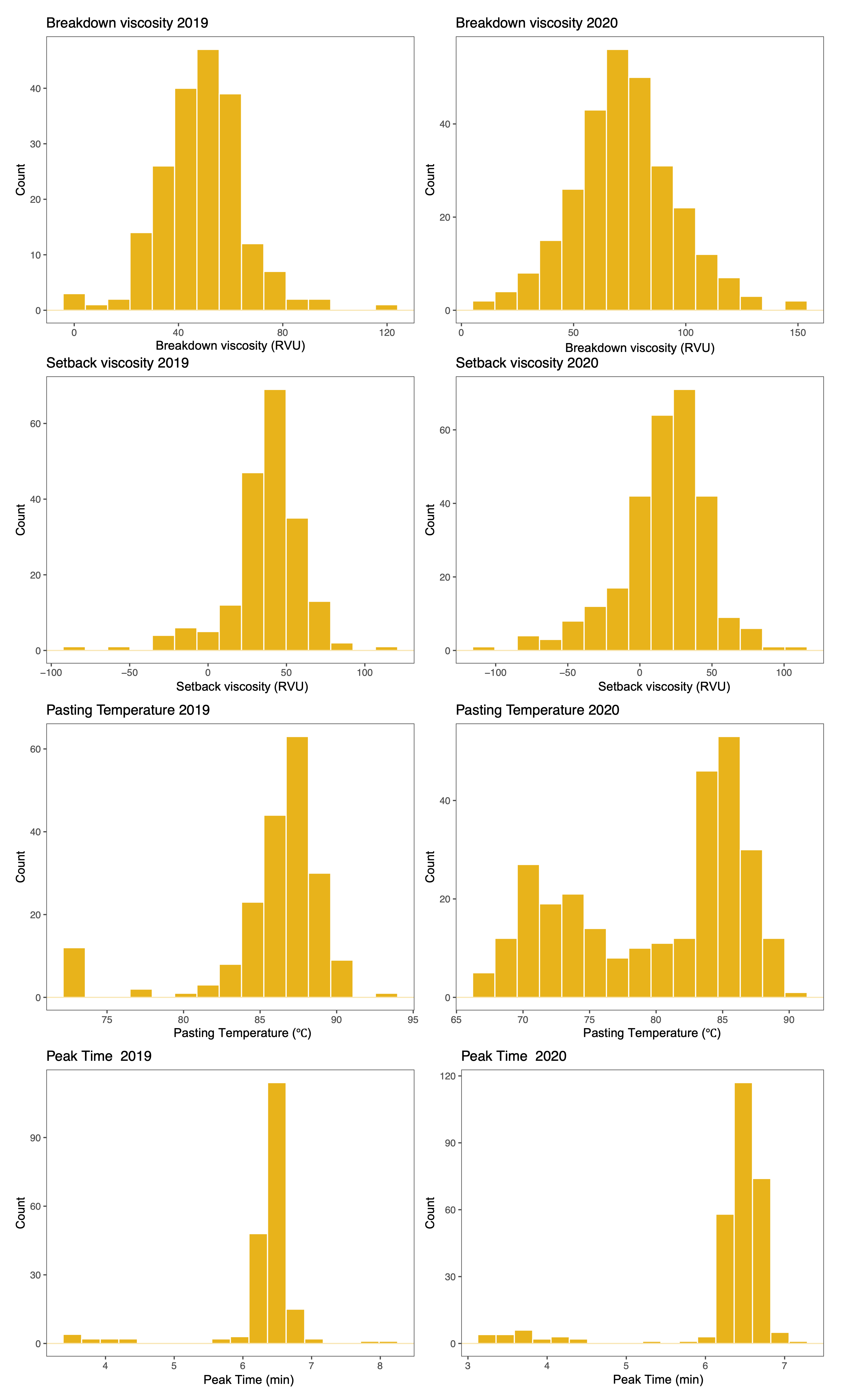

Supplement: Supplementary file 2 — Supplementary Material 2: Figure S1 Histogram of other starch pasting properties. Including breakdown viscosity, setback viscosity, pasting temperature and peak time in 2019 and 2020 each. [file 12284_2025_782_MOESM2_ESM.png]

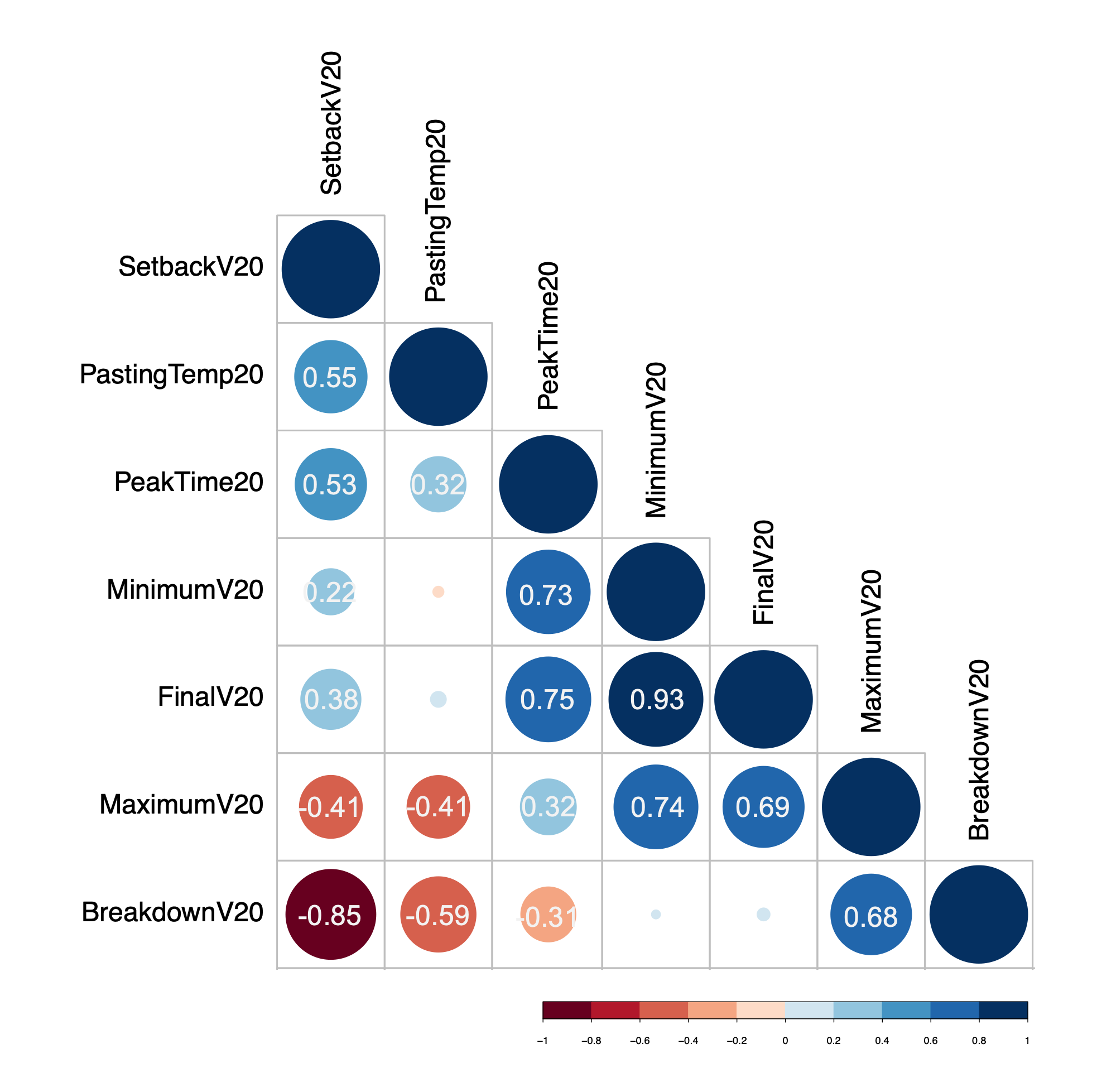

Supplement: Supplementary file 3 — Supplementary Material 3: Figure S2 Correlation analysis of the starch pasting properties measured from RVA in 2020. Circle size imply the strength of correlation, while color indicate blue as positive correlation and red as negative correlation between traits. Correlation coefficients with a significant difference of at most 0.05 are marked with the values. V stands for viscosity. [file 12284_2025_782_MOESM3_ESM.png]

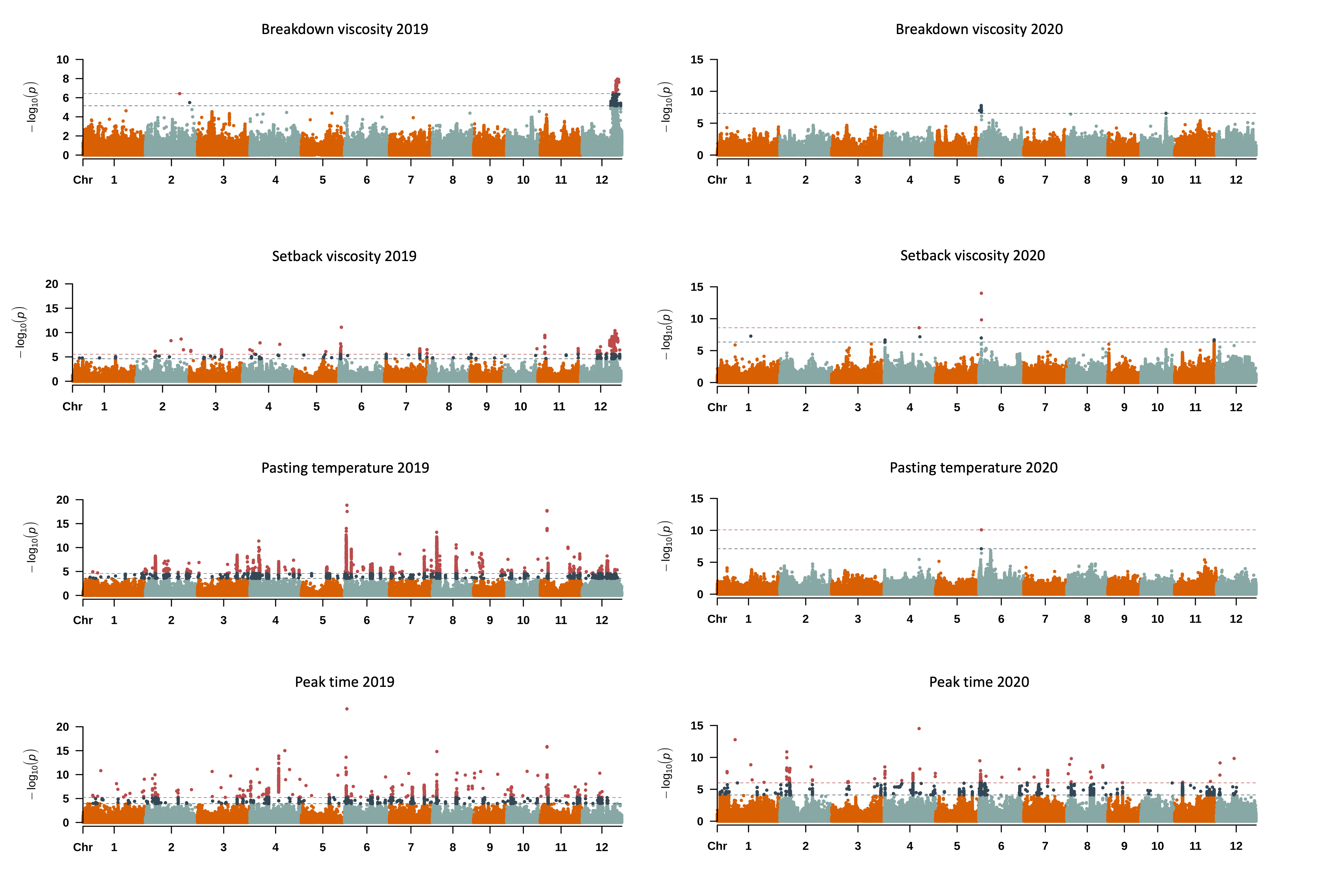

Supplement: Supplementary file 4 — Supplementary Material 4: Figure S3 Manhattan plots of other starch pasting properties. Including breakdown viscosity, setback viscosity, pasting temperature and peak time in 2019 and 2020 each. The blue dots above blue dashed lines indicate the SNPs above the lowest false discovery rate adjusted P-values were 0.05. The red dots above red dashed lines indicate < the threshold of 0.01. [file 12284_2025_782_MOESM4_ESM.png]

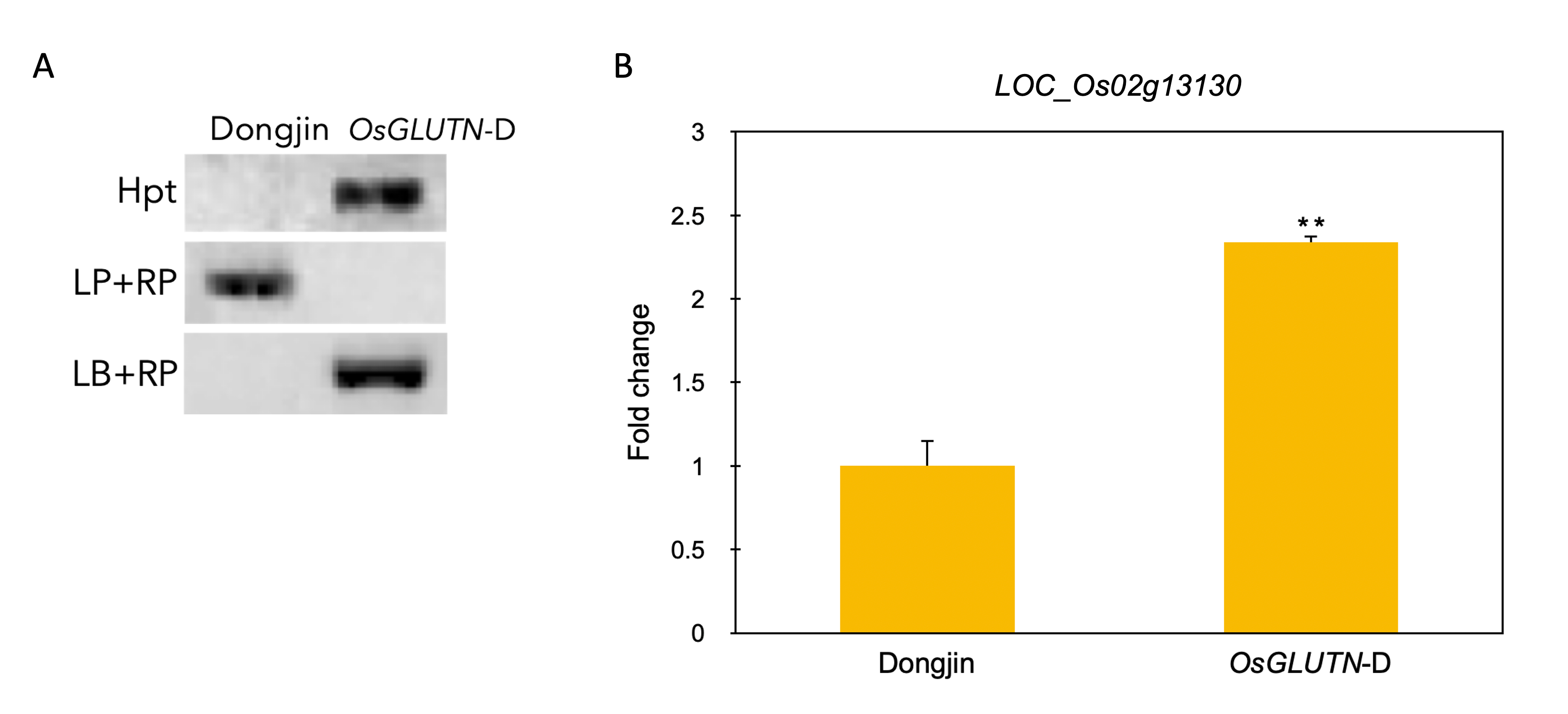

Supplement: Supplementary file 5 — Supplementary Material 5: Figure S4 PCR and qRT-PCR of T-DNA line. (A) PCR confirming the T-DNA insertion, LP, RP, and LB stands for left gene specific primer, right gene specific primer, and pGA2715 left border primer, respectively. The 35S enhancer active tagging line, OsGLUTN-D, was compared with its wild type parental cultivar, Dongjin. (B) Relative gene expression level of wild type, Dongjin, and OsGLUTN-D. Significance level indicates **, P < 0.01. [file 12284_2025_782_MOESM5_ESM.png]

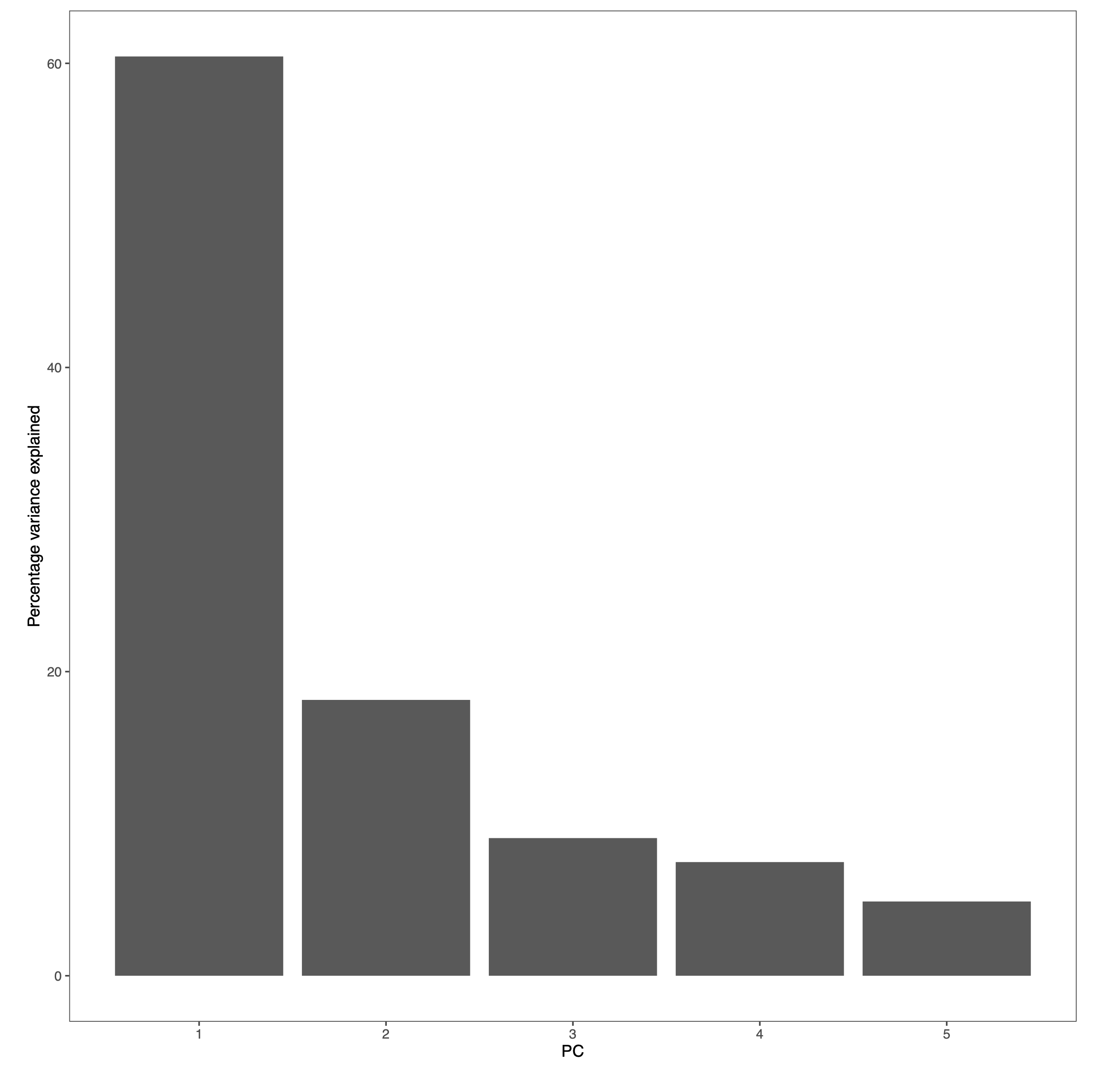

Supplement: Supplementary file 6 — Supplementary Material 6: Figure S5 Explained variances of the principal components. Contributing PCs on the x axis, and explained variances (%) on the y axis of the bar graph. [file 12284_2025_782_MOESM6_ESM.png]

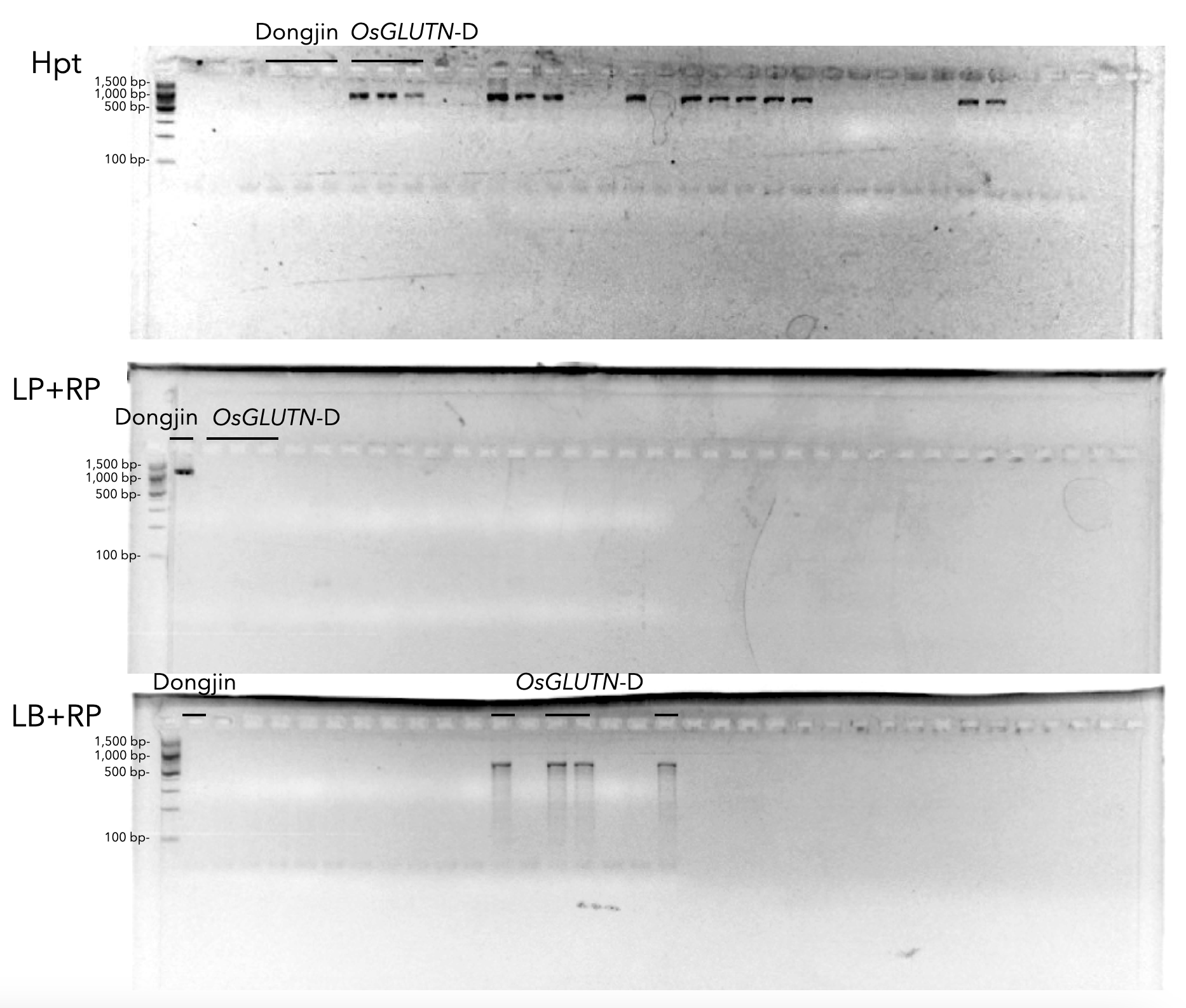

Supplement: Supplementary file 7 — Supplementary Material 7 [file 12284_2025_782_MOESM7_ESM.png]
